# The transcriptional regulatory network in the drought response and its crosstalk in abiotic stress responses including drought, cold, and heat

**DOI:** 10.3389/fpls.2014.00170

**Published:** 2014-05-16

**Authors:** Kazuo Nakashima, Kazuko Yamaguchi-Shinozaki, Kazuo Shinozaki

**Affiliations:** ^1^Biological Resources and Post-harvest Division, Japan International Research Center for Agricultural SciencesTsukuba, Japan; ^2^Laboratory of Plant Molecular Physiology, Graduate School of Agricultural and Life Sciences, The University of TokyoTokyo, Japan; ^3^Gene Discovery Research Group, RIKEN Center for Sustainable Resource ScienceYokohama, Japan

**Keywords:** ABA, transcription factor, signal transduction, abiotic stress, drought

## Abstract

Drought negatively impacts plant growth and the productivity of crops around the world. Understanding the molecular mechanisms in the drought response is important for improvement of drought tolerance using molecular techniques. In plants, abscisic acid (ABA) is accumulated under osmotic stress conditions caused by drought, and has a key role in stress responses and tolerance. Comprehensive molecular analyses have shown that ABA regulates the expression of many genes under osmotic stress conditions, and the ABA-responsive element (ABRE) is the major *cis*-element for ABA-responsive gene expression. Transcription factors (TFs) are master regulators of gene expression. ABRE-binding protein and ABRE-binding factor TFs control gene expression in an ABA-dependent manner. SNF1-related protein kinases 2, group A 2C-type protein phosphatases, and ABA receptors were shown to control the ABA signaling pathway. ABA-independent signaling pathways such as dehydration-responsive element-binding protein TFs and NAC TFs are also involved in stress responses including drought, heat, and cold. Recent studies have suggested that there are interactions between the major ABA signaling pathway and other signaling factors in stress responses. The important roles of these TFs in crosstalk among abiotic stress responses will be discussed. Control of ABA or stress signaling factor expression can improve tolerance to environmental stresses. Recent studies using crops have shown that stress-specific overexpression of TFs improves drought tolerance and grain yield compared with controls in the field.

## INTRODUCTION

**FIGURE 1:**
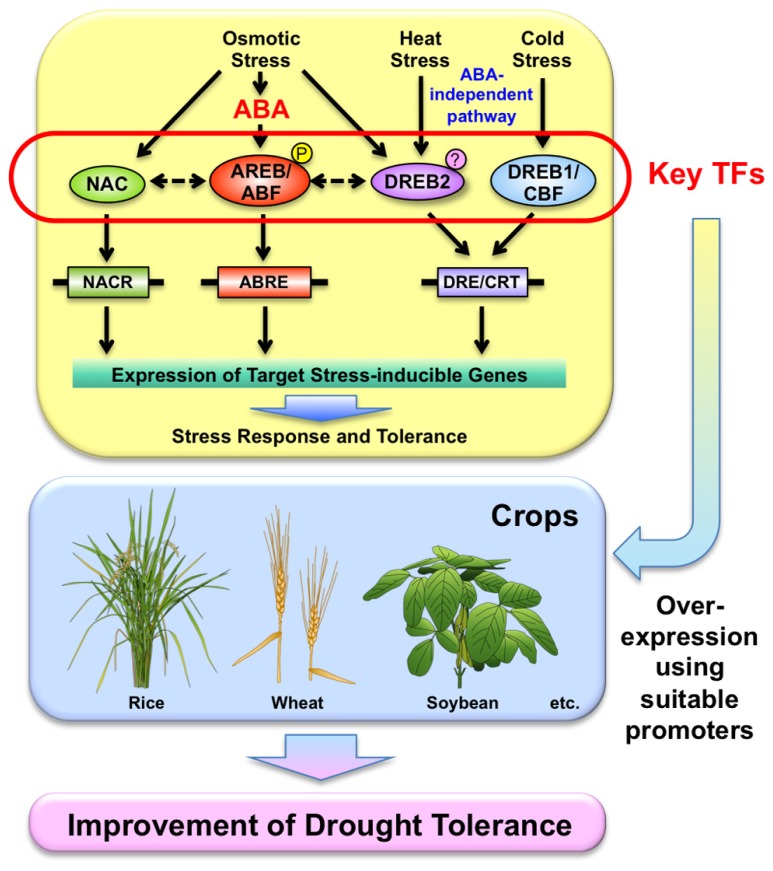
**Utilization of transcription factors (TFs) involved in stress-responsive pathways in stress responses for the improvement of drought tolerance of crops.** Usage of suitable promoters might be necessary to control their expression.

The world population is expected to reach nine billion by 2050. Considering this population increase, crop yields need to be improved by 40% in areas where drought is likely to occur by 2025 (Pennisi, 2008). In addition, frequent occurrences of drought and abnormal weather events have lately been observed all over the world. Drought negatively impacts plant growth and crop production (Bray et al., 2000). Almost every year, some region of the earth is hit by drought, damaging crops, and disrupting agricultural production. Severe drought affected the central and south of the US Corn Belt during 2012 (Edmeades, 2013). Drought also causes great damage to the production of other crops such as rice, wheat, and soybean. The southern states of Brazil, which account for 40% of the soybean production by the second leading producer worldwide, lost more than 20% of their production because of drought during the 2003/2004 and 2004/2005 seasons (Polizel et al., 2011). The development of stress-tolerant crops will be significantly advantageous in areas where such stresses occur frequently. Recently, some progress has been made toward identification of stress-related genes potentially capable of increasing the tolerance of plants to abiotic stress. Understanding the molecular mechanisms in the drought response is important to improve drought tolerance using molecular techniques. ABA accumulates under osmotic stress caused by drought, but also by other water limiting conditions, and plays an important role in stress responses and tolerance in plants (reviewed in Finkelstein et al., 2002; Yamaguchi-Shinozaki and Shinozaki, 2006; Nakashima et al., 2009b; **Figure 1**). Molecular studies have revealed that ABA-independent gene expression is also important in stress tolerance in plants (**Figure 1**). In this review, we summarize some of the most important TFs in drought responses and discuss their regulatory networks and crosstalk in abiotic stress responses. By applying current knowledge of stress-regulated TFs and their target genes, improvement of drought stress tolerance is in progress in various crops using transgenic technology.

## AREB/ABF TFs FOR ABA-DEPENDENT GENE EXPRESSION

The promoter regions of ABA-responsive genes contain a conserved *cis*-element, named the ABRE (PyACGTGG/TC), which controls gene expression (**Figure 1**). Studies have revealed that expression of ABA-responsive genes requires more than one ABRE or a combination of an ABRE and a CE for a functional promoter (reviewed in Fujita et al., 2011, 2013; Nakashima and Yamaguchi-Shinozaki, 2013). Comprehensive and molecular analyses showed that ABA regulates the expression of many genes under osmotic stress conditions, and that the ABRE is the major *cis*-element for ABA-responsive gene expression (Maruyama et al., 2012). AREB/ABFs are bZIP TFs that regulate ABA-dependent gene expression, acting as major TFs under abiotic stress conditions in *Arabidopsis* (reviewed in Fujita et al., 2011, 2013; **Figure 1**). Among the nine members of the AREB/ABF TF family identified in *Arabidopsis*, AREB1/ABF2 has been reported to control ABA signaling and environmental stress responses during the vegetative growth stage. The AREB/ABF TFs are induced by abiotic stress and their transcriptional activities are controlled by ABA-dependent phosphorylation. ABA is required for full activation of AREB1 (Fujita et al., 2005; Yoshida et al., 2010) and its activity is regulated by the ABA-dependent phosphorylation of multiple sites within conserved domains (Furihata et al., 2006). Transgenic *Arabidopsis* plants overexpressing deleted and active forms of AREB1 showed enhanced drought tolerance and ABA hypersensitivity (Fujita et al., 2005). Overexpression of *AREB1* also improved drought tolerance in rice and soybean (Oh et al., 2005; Barbosa et al., 2013). Progress in understanding ABA perception and signal transduction has been made recently (reviewed in Cutler et al., 2010; Raghavendra et al., 2010; Umezawa et al., 2010; Weiner et al., 2010; Nakashima and Yamaguchi-Shinozaki, 2013). It was revealed that SnRK2, group A PP2Cs, and RCAR/PYR/PYL ABA receptors control the ABA signaling pathway including AREB/ABFs in land plants (reviewed in Umezawa et al., 2010; Miyakawa et al., 2013; Nakashima and Yamaguchi-Shinozaki, 2013). The phosphorylation of AREB/ABFs by SnRK2s is critical in the ABA-dependent signaling network (Fujita et al., 2009; Nakashima et al., 2009a; Umezawa et al., 2013). Recent studies have indicated that group A PP2Cs evolved early in land plants as key regulators of intrinsic desiccation tolerance, such as in the moss *Physcomitrella patens* (Komatsu et al., 2013). Perception and signaling factors such as PYL4 can also be used to improve stress tolerance (Pizzio et al., 2013).

## DREB1/CBF TFs FOR COLD-RESPONSIVE GENE EXPRESSION TO IMPROVE DROUGHT TOLERANCE

Analysis of the promoter regions of genes showing ABA-independent expression in stress responses and tolerance has shown a *cis*-element with the sequence A/GCCGAC, designated the DRE/CRT (**Figure 1**). Two groups of AP2/ERF TFs were identified as DREB; DREB1/CBF and DREB2 in *Arabidopsis* (Liu et al., 1998). DREB1/CBF TFs specifically interact with the DRE/CRT and control the expression of a large number of stress-responsive genes in *Arabidopsis*. Improvements in tolerance to drought, salinity and freezing stresses have been reported in transgenic *Arabidopsis* overexpressing DREB1/CBF TFs, although their constitutive expression causes growth defects (Liu et al., 1998; Kasuga et al., 1999). However, overexpression of *DREB1* under the control of the *Arabidopsis* stress-responsive *RD29A* promoter improved stress tolerance in *Arabidopsis* without growth defects (Kasuga et al., 1999). Cold-inducible *DREB1/CBF* genes have also been isolated from a number of plant species, such as maize, oilseed rape, rye (*Secale cereale*), rice, tomato, and wheat (*Triticum aestivum*; reviewed in Mizoi et al., 2012). Interestingly, the major QTLs for tolerance to frost in *Arabidopsis*, diploid wheat (*T. monococcum*) and barley map to *DREB1/CBF* genes, and the expression levels of *DREB1/CBF* genes are correlated with frost tolerance (Vágújfalvi et al., 2003; Alonso-Blanco et al., 2005; Francia et al., 2007; Knox et al., 2008). Thus, the function of the DREB1/CBF regulon in the regulation of cold stress responses is widely conserved in angiosperms. Overexpression of DREB/CBF TFs has been reported to enhance drought tolerance in transgenic crops including chrysanthemum (Hong et al., 2006), peanut (Bhatnagar-Mathur et al., 2007, Bhatnagar-Mathur et al., 2013), potato (Behnam et al., 2007; Iwaki et al., 2013), rice (Oh et al., 2005; Ito et al., 2006; Datta et al., 2012), soybean (Polizel et al., 2011; de Paiva Rolla et al., 2013), tobacco (Kasuga et al., 2004), tomato (Hsieh et al., 2002a,b), and wheat (Pellegrineschi et al., 2004; Saint Pierre et al., 2012). For example, rice DREB1/CBF-type TFs involved in cold-responsive gene expression also conferred improved tolerance to drought in transgenic rice (Ito et al., 2006). The rice *DREB1/CBF*-type genes, *OsDREB1A* and *OsDREB1B*, are induced by cold stress. Transgenic *Arabidopsis* and rice plants overexpressing rice *OsDREB1* or *Arabidopsis DREB1* genes showed improved tolerance to drought, high-salt and cold stresses but defective growth under normal growth conditions. Elevated contents of osmoprotectants including free proline and soluble sugars were detected in the transgenic rice. These results indicate that the *DREB1/CBF* regulon is conserved in rice, and that DREB1/CBF-type genes may be useful for improvement of tolerance to different environmental stresses in various kinds of transgenic monocot plants as well as dicot plants.

## DREB2 TFs FOR OSMOTIC- AND HEAT-RESPONSIVE GENE EXPRESSION TO IMPROVE DROUGHT TOLERANCE

The *DREB2* gene encoding a DRE/CRT-binding protein is induced by osmotic stress (Liu et al., 1998; **Figure 1**). However, transgenic plants overexpressing *DREB2A* did not show any changes in phenotype. Domain analysis of DREB2A using *Arabidopsis* protoplasts showed that deletion of the central region makes DREB2A constitutively active (DREB2Aca), indicating that this region contains a negative regulatory domain (NRD; Sakuma et al., 2006a). Overexpression of *DREB2Aca* induced growth defects, up-regulation of stress-inducible genes, and enhanced drought tolerance (Sakuma et al., 2006a). Stress-inducible overexpression of *DREB2ca* improved drought tolerance in *Arabidopsis* and soybean without growth defects (Sakuma et al., 2006a; Engels et al., 2013). The NRD region of DREB2A is required for regulation of DREB2A protein stability. As mentioned above, overexpression of *DREB1A* improves freezing and dehydration stress tolerance in transgenic plants. By contrast, overexpression of *DREB2Aca* improves dehydration stress tolerance but only slightly improves freezing stress tolerance in transgenic plants. Integrated analysis of transcripts and metabolites was conducted to see the difference in the downstream gene products of DREB1A and DREB2A in *Arabidopsis* (Maruyama et al., 2009). Microarray analysis indicated that the downstream gene products of DREB1A and those of DREB2A have similar putative functions, but the expression of genes for carbohydrate metabolism in *DREB1A* and *DREB2A* transgenic plants is very different. Under dehydration and cold conditions, expression of genes for starch-degradation, sucrose metabolism and sugar alcohol synthesis changes dynamically. As a result, many kinds of mono-, di-, and trisaccharides, and sugar alcohols accumulate in plants. Overexpression of *DREB1A* caused similar changes in these metabolic processes, and these changes might improve dehydration and freezing stress tolerance in transgenic plants. By contrast, overexpression of *DREB2Aca* did not increase the level of these metabolites in transgenic plants. In addition, degradation of DREB2A is mediated by DRIPs, which are C3HC4 RING domain-containing proteins. DRIPs bind to DREB2A and function as E3 ubiquitin ligases mediating ubiquitination of DREB2A (Qin et al., 2008). Overexpression of *DREB2Aca* also induced expression of genes related to heat shock stress and improved thermotolerance in transgenic plants (Sakuma et al., 2006b). These results indicate that DREB2s function in both dehydration and heat shock stress responses. DREB2-type proteins have been isolated from a number of other plant species such as barley, rice, sunflower, maize, and wheat (Mizoi et al., 2012). GmDREB2A;2 is a DREB2A ortholog in soybean (Mizoi et al., 2013), but there are differences between DREB2A and GmDREB2A;2 in the NRD sequence. The effects on gene expression in transgenic plants overexpressing *GmDREB2A;2* are different from those in transgenic plants overexpressing* DREB2A*. This suggests that specialization in DREB2 regulons has occurred, although their basic functions are conserved between *Arabidopsis* and soybean. Recently, GWAS of ZmDREB2 and natural variations in the drought tolerance of maize (*Zea mays*) indicated that natural variation in the promoter region of *ZmDREB2.7* contributes to drought tolerance in maize (Liu et al., 2013). The favorable* ZmDREB2.7* allele may be a good resource for improving drought tolerance in maize. Recent studies suggest that DREB2 has important functions in drought tolerance, and that it can be used for improvement of drought tolerance in crops.

## NAC TFs FOR DROUGHT-RESPONSIVE GENE EXPRESSION TO IMPROVE DROUGHT TOLERANCE

NAM, ATAF, and CUC TF proteins are plant-specific TFs. More than 100 *NAC* genes have been identified in *Arabidopsis* and rice (reviewed in Nakashima et al., 2012). Phylogenetic analyses indicate that six groups were established in an ancient moss. NAC TFs have a variety of important functions in development and stress responses. The genes in the SNAC group have important roles in the control of environmental stress tolerance (reviewed in Nakashima et al., 2012; **Figure 1**), and can bind to the NACR (NAC recognition sequence; CACG core). Stress-responsive *Arabidopsis SNAC* genes such as *RD26* and *ATAF1*, and rice *SNAC* genes such as *SNAC1*, *OsNAC6*/*SNAC2*, and *OsNAC5* can improve drought and/or high-salt stress tolerance when overexpressed (Tran et al., 2004; Hu et al., 2006; Nakashima et al., 2007; Takasaki et al., 2010; reviewed in Nakashima et al., 2012). Stress-responsive overexpression of *NACs* utilizing rice stress-responsive *LIP9*, *OsNAC6*, or *OsHox24* promoters is effective in inducing stress tolerance without the inhibitory effects of NAC on plant growth (Nakashima et al., 2007, 2012, 2014; Takasaki et al., 2010). Recent studies have suggested that the root-specific promoter *RCc3* is useful for the overexpression of *SNACs* such as *SNAC1* and *OsNAC10* to enhance the abiotic stress tolerance of rice in field conditions (Jeong et al., 2010, 2013; Redillas et al., 2012). These results indicate that SNACs have important roles in the control of abiotic stress responses and tolerance and that it is possible to improve stress tolerance by overexpressing SNACs using suitable promoters in the field. The many kinds of drought-responsive or tissue/organ-specific promoters reported for roots and stomata might be effective tools to control the expression of drought-responsive factors that cause growth defects at the right time and right position (Nakashima et al., 2007, 2014; Rai et al., 2009; Wu et al., 2009; Xiao et al., 2009; Yi et al., 2010; Ganguly et al., 2011; Yang and Xiong, 2011; Bang et al., 2013; Rusconi et al., 2013).

## INTERACTIONS BETWEEN MULTIPLE TFs IN DROUGHT RESPONSES

Evidence for interaction between the AREB/ABFs and DREB/CBFs has been reported. The DRE/CRT motif in the promoters of drought-responsive genes is a binding region for an ABA-independent DREB/CBF TF and functions as a CE for ABRE in ABA-dependent gene expression (Narusaka et al., 2003). Lee et al. (2010) showed that the DREB1A/CBF3, DREB2A, and DREB2C proteins interact physically with AREB/ABF proteins. These data suggest crosstalk between elements of the ABA-dependent and -independent response pathways. Moreover, interactions in the signaling pathways have also been indicated. Kim et al. (2011) reported that an ABRE promoter sequence, AREB/ABF TFs, and SnRK2s are involved in expression of the *DREB2A* gene under osmotic stress conditions, suggesting complex interaction between the AREB and DREB regulons at the gene expression level as well as the protein level.

Interaction between the AREB/ABFs and NACs has also been indicated at the gene expression level. Jensen et al. (2013) reported that *Arabidopsis* SNAC TF ATAF1 directly regulates the ABA biosynthetic gene *NCED3* in *Arabidopsis*, suggesting that SNAC TFs may regulate ABA-dependent gene expression of ABRE regulons. On the other hand, the promoters of *SNAC* genes contain ABRE sequences (Nakashima et al., 2012). Recently, Xu et al. (2013) reported that *Arabidopsis* ANAC096 cooperates with AREB/ABF factors (ABF2/AREB1 and ABF4/AREB2) in dehydration and osmotic stress responses. These results indicate complex interaction between the AREB/ABF and NAC regulons.

Finally, interaction between DREB/CBFs and other kinds of AP2/ERFs at the gene expression level has also been suggested. Cheng et al. (2013) reported that the *Arabidopsis* ERF1 regulates gene expression by binding to two kinds of *cis*-elements, the GCC box and DRE/CRT, in response to different stress signals. ERF1 is an upstream TF in both ethylene and jasmonate signaling and is involved in resistance to pathogens. Their results suggested that ERF1 bound to the GCC box but not the DRE/CRT in response to biotic stress, and to the DRE/CRT under abiotic stress. These results suggest that ERF1 may integrate ethylene, jasmonate, and ABA signaling and play an important role in biotic and abiotic stress responses.

## CONCLUSION

Molecular analysis has suggested that drought-responsive TFs such as DREB1/CBF, DREB2, AREB/ABF, and NAC TFs function in drought responses and tolerance (**Figure 1**). These TFs also function in crosstalk in abiotic stress responses, such as drought, cold, and heat. As mentioned above, these factors can be used to improve drought tolerance in a variety of crops. Our group has utilized these key TFs for the improvement of drought tolerance in crops including rice, wheat, and soybean in collaboration with international and domestic institutes (Pellegrineschi et al., 2004; Hong et al., 2006; Behnam et al., 2007; Bhatnagar-Mathur et al., 2007; Polizel et al., 2011; Datta et al., 2012; Ishizaki et al., 2012; Saint Pierre et al., 2012; Barbosa et al., 2013; Bhatnagar-Mathur et al., 2013; de Paiva Rolla et al., 2013; Engels et al., 2013; Iwaki et al., 2013). Some results using crops including rice and peanut have shown that stress-specific overexpression of *DREB1A* improves drought tolerance and grain yield compared with controls in the field (Datta et al., 2012; Bhatnagar-Mathur et al., 2013). These results suggest that overexpression of key TFs under the control of suitable promoters can improve stress tolerance, although the regulatory network in the plant response is complex in water limiting environments (**Figure 1**). Since TFs function in balanced crosstalk in abiotic stress responses, overexpression of a certain TF may affect other signaling pathways. Thus, we should examine the molecular effects of overexpressing TFs in addition to conducting stress tolerance assays. In addition, the effects of a transgene may depend on the genetic background of the species or cultivar used for transformation. Furthermore, since the degree of drought varies in actual fields (strength, timing, and period of stress, complex stresses such as drought with heat stress etc.), the effect of a transgene may differ depending on environmental conditions. Continuous field experiments might be necessary to see the effects of transgene-encoded TFs in the field using a variety of genotypes and environments. Recently, QTL analyses have revealed novel genes involved in drought resistance. *DEEPER ROOTING 1* (*DRO1*), a QTL controlling root growth angle in rice, was cloned and characterized (Uga et al., 2013). This study revealed that changes in root system architecture can improve drought avoidance. Other drought resistant QTLs have also been reported in rice. Multiple QTLs were reported in the rice mega-variety IR64 that enhance the yield under drought conditions (Swamy et al., 2013). Combinations/pyramiding of transgenic plants and QTL drought resistant varieties by marker-assist selection (MAS) may promote drought tolerance.

## Conflict of Interest Statement

The authors declare that the research was conducted in the absence of any commercial or financial relationships that could be construed as a potential conflict of interest.

## ABBREVIATIONS

ABA: abscisic acid
ABF: ABRE-binding factor
ABRE: ABA-responsive element
AP2: APETALA 2
AREB: ABRE-binding protein
bZIP: basic leucine zipper
CBF: CRT binding factor
CE: coupling element
DRE: dehydration-responsive element
DREB: DRE-binding protein
DRIP: DREB2A-interacting protein
CRT: C-repeat
ERF: ethylene-responsive element binding factor
GWAS: genome-wide association study
NAC: NAM, ATAF, and CUC
PP2C: 2C-type protein phosphatase
PYL: PYR1-like
PYR: pyrabactin resistance
QTL: quantitative trait locus
RCAR: regulatory component of ABA receptor
SNAC: stress-responsive NAC
SnRK2: SNF1-related protein kinase 2
TF: transcription factor.
